# A Breach of Ethics

**Published:** 1889-07

**Authors:** 


**There** is no more villainous nostrum on the market than the so-called "Microbe Killer" "*de* Radam." It is a "patent medicine" of the most brazen stamp, gotten up by a man who is totally ignorant of chemistry, therapeutics, pathology, and everything connected with the practice of medicine—a gardener and florist. But it is advertised in the pages of a *Medical Journal*, which, of all medical journals in the world should, to be consistent, have refused it space. A journal, edited by a committee or board representing the Chicago *Post Graduate Medical College*, the official journal of that institute, of which institution, we learn, the honorable and venerable "father in (medical) Israel"—Dr. F. S. Davis—is a director.

If this is not a breach, and a most flagrant breach, of medical and journalistic ethics, what is? If the pages of *such* journals are to be thrown open to *such* quack remedies, farewell, and a long farewell, to ethical and legitimate medical journalism. For very shame, brothers.

